# Editorial: Focusing on T-Cells for Novel Treatments of Systemic Lupus Erythematosus

**DOI:** 10.3389/fimmu.2021.744866

**Published:** 2021-08-06

**Authors:** Kunihiro Ichinose, Christian Michael Hedrich, Vaishali R. Moulton, Masayuki Mizui

**Affiliations:** ^1^Department of Immunology and Rheumatology, Division of Advanced Preventive Medical Sciences, Nagasaki University Graduate School of Biomedical Sciences, Nagasaki, Japan; ^2^Department of Women’s and Children’s Health, Institute of Life Course and Medical Sciences, University of Liverpool, Liverpool, United Kingdom; ^3^Division of Rheumatology and Clinical Immunology, Alder Hey Children’s NHS Foundation Trust Hospital, Liverpool, United Kingdom; ^4^Division of Rheumatology and Clinical Immunology, Department of Medicine, Beth Israel Deaconess Medical Center and Harvard Medical School, Boston, MA, United States; ^5^Department of Nephrology, Graduate School of Medicine, Osaka University, Suita, Japan

**Keywords:** lupus T-cells, immunometabolism, innate lymphocytes, post-transcriptional regulation, Th17/Treg balance

The pathogenesis of Systemic Lupus Erythematosus (SLE) involves all components of the immune system, including immune cells (T cells, B cells, antigen presenting cells), autoantibody production and immune-complex deposition. As autoantibody-mediated inflammation and damage are downstream events in SLE pathophysiology, investigating molecular mechanisms driving immune cell alterations will deliver disease mechanisms and treatment targets.

Dysregulated immune responses to self- and foreign antigens can cause and/or amplify multi-organ complications. This is addressed by the manuscript from Spihlman et al. who review clinical and immunological parallels between COVID-19 and SLE. Immune responses against self-antigens in SLE have much in common with those against SARS-CoV-2, and a variety of therapeutic approaches, including the use of corticosteroids and immunosuppressive agents, are effective in both. Thus, understanding immune dysregulation in SLE may aid in the treatment of COVID-19 and vice versa.

This Research Topic ‘Focusing on T-cells for novel treatments of SLE’ focuses on summarizing current knowledge on lymphocyte dysregulation in SLE, centering on T-cells. Over recent years, lupus-prone mice have improved our understanding of SLE in humans, including the involvement of effector CD3^+^CD4^+^ and CD3^+^CD4^-^CD8^-^, so-called “double-negative” (DN), T cells. Although numerically expanded DN T cell populations are a characteristic of lupus in mice and humans, their exact roles and origin remain controversial (1). Liu et al. found that CD138 (Syndecan-1) positive DN T cells, dominantly derived from CD4^+^ T cells, have a central memory phenotype and are involved in the activation of autoreactive B cells in the MRL/lpr mouse, a fulminant mice lupus model. By contrast, Flores-Mendoza et al. showed that DN T cells were induced only when Fas/FasL-deprived CD8^+^ T cells were re-stimulated with self-antigens. Furthermore, Fas/FasL on CD8^+^ T cells is involved in the retention of CD8 expression after antigen re-stimulation. Therefore, loss of CD8 (or CD4) expression may limit excessive immune responses. However, as they express high levels of effector cytokines, DN T cells may also exacerbate and/or maintain autoimmunity (1, 2). Further studies are warranted to elucidate the exact role and function of DN T cells in autoimmune/inflammatory disease.

The importance of CD3^+^CD4^+^ Th17 cells in lupus pathogenesis is widely accepted. Usually, to maintain immune tolerance, the development of Th17 is in balance with that of regulatory T cells (Treg), especially in the intestinal tract (3). In autoimmune/inflammatory disease, such as SLE, the Th17/Treg imbalance may be altered and contribute to the immune pathology. As reviewed by Koga et al., molecules such as Protein phosphatase 2A (PP2A), Calcium/calmodulin kinase IV (CaMK4), and cAMP-responsive element modulator (CREM)/CREM inducible cAMP early repressor (ICER), which are upregulated in T cells of SLE patients, are important for Th17 differentiation and involved in the development of SLE in mice. On the other hand, IL-17A-deficient lupus-prone mice and animals treated with anti-IL-17A antibodies still develop lupus (4). Thus, the exact role of IL-17A and Th17 cells, and the balance between Th17 and Treg populations in SLE pathophysiology remains unclear.

Indeed, the balance between Th17 and Treg cells, and mechanisms controlling their balance, including immunometabolism, is currently in the focus of research and reviewed by Shan et al. in this topic. Glycolysis and lipid synthesis are required for Th17 differentiation and inhibition of these metabolic signals alleviates disease activity in lupus-prone mice and SLE patients. Furthermore, Kono et al. described the importance of amino acid metabolism, including glutaminolysis, for lymphocyte activation and differentiation. Amino acid availability is crucial for mechanistic target of rapamycin (mTOR) activation, which is required for Th17 differentiation (5). The Nuclear factor erythroid 2-related factor 2(NRF2)/Kelch ECH associating protein 1(Keap1) pathway, a critical regulator of the antioxidant system and REDOX metabolism, is also involved in lupus pathogenesis. NRF2 deficient female mice are prone to develop lupus nephritis (6). Lupus-prone B6/lpr lacking NRF2 aggravates glomerulonephritis with increased numbers of Th17 cells (7). Indeed, dimethylfumalate, an activator of the NRF2 pathway, is now widely used for the treatment of multiple sclerosis, another Th17-driven autoimmune/inflammatory disease. In their review in this special topic, Ohl et al. discuss the possibility of targeting NRF2/Keap1 for the treatment of SLE.

In this special topic, Iwata et al. investigated fatty acid synthesis in T cells from SLE patients, and identified alterations in Th1 subsets of SLE patients and their involvement in disease pathology. Authors dissected the role of lipid metabolism in the induction of inflammatory subsets of Th1 cells, including inhibition of fatty acid synthesis that effectively altered the phenotype of peripheral T cells in SLE, while rapamycin was not as effective. While T-bet^hi^CXCR3^lo^ effector cells and T-bet^+^Foxp3^lo^ non-suppressor cells (which produce large amounts of IFN-γ) are abundant in SLE, T-bet^+^Foxp3^hi^ activated Treg cells (which do not produce IFN-γ) are lacking. These changes may be involved in the therapeutic resistance as treatment of stimulated memory CD4^+^ T cells with rapamycin and 2-deoxy-D-glucose (2DG) suppressed T-bet^+^Foxp3^-^ cells *in vitro* and induced T-bet^+^Foxp3^+^(lo/hi) cells. Interestingly, rapamycin alone enhanced lipid metabolism and induced IFN-γ-producing T-bet^+^Foxp3^lo^ cells, while 2DG induced non-IFN-γ-producing T-bet^+^Foxp3^hi^ cells. In memory CD4^+^ cells from SLE patients, inhibition of fatty acid synthesis suppressed IFN-γ production and enhanced Foxp3 expression in T-bet^+^Foxp3^+^ cells. Thus, in SLE, metabolic abnormalities, such as enhanced fatty acid synthesis, contribute to the overproduction of IFN-γ by Th1 cells and an imbalance of Th1 subsets.

Innate lymphocytes and natural killer cells (NK) are lymphocytic cells that cannot solely be attributed to the innate or adaptive immune system. An involvement of innate lymphocytes in lupus pathology has emerged recently (8). Among three sub-types of innate lymphoid cells (ILCs), Hu et al. reported that type 3 ILCs (ILC3s) produce IL-22 in the kidney of lupus-prone MRL/lpr mice and induce chemokine expression in tubular epithelial cells. As this is the first report investigating ILC3 in the kidney, further research is warranted. Humbel et al. investigate the role of NK cells in SLE. Authors report that NK cell numbers in the peripheral blood is reduced in SLE patients, and analyzed an array of surface markers, including SLAMF1, SLAMF7 and CD38. The expression of CD38 is increased lupus NK cells. Both the anti-SLAMF7 antibody elotuzumab and the anti-CD38 antibody daratumumab can enhance NK cell function in SLE. Notably, anti-CD38 was recently reported to be effective for the treatment of SLE (9), therefore the involvement of NK cells in anti-CD38 treatment should be considered.

Lastly, evidence is accumulating to suggest that post-transcriptional regulators, such as micro-RNAs, other non-coding RNAs, and RNA-binding proteins, play a critical role in lupus pathogenesis (10, 11). Here, Hiramatsu-Asano et al. focused on miR-223-3p, which is upregulated in lupus-prone mice, and found that it regulates the expression of sphingosine-1-phosphate receptor (S1PR1), a pivotal receptor for peripheral T cell circulation. Authors show that loss of miR223 exacerbates the lupus phenotype by increasing the population of S1PR1^+^CD4^+^ T cells and promoting their infiltration into inflamed kidney tissue. In addition to regulating S1PR1 function, miR223 may be effective for SLE treatment by targeting S1PR1^+^CD4^+^ T cells. Since both SLE-specific upregulation and downregulation of miRNAs are potential therapeutic targets, more reports and targets are expected in the future.

Excellent reports were provided covering a wide spectrum of research areas in SLE, including immunometabolism, innate lymphocyte biology and function, post-transcriptional regulation, and basic T-cell biology. The central role of T-cells in SLE is underscored by reports on voclosporin, a T-cell targeted calcineurin inhibitor, to be effective in the treatment of lupus nephritis (12). The current collection of manuscripts and future reports will help us to better understand the pathophysiology of SLE. Novel molecular and cellular candidates proposed in this special topic are expected to be further investigated for their suitability as biomarkers and/or treatment targets in SLE.

## Author Contributions

KI and MM wrote the first draft of the editorial. This was revised by CH and VM with valuable comments and suggestions. All authors contributed to the article and approved the submitted version.

## Conflict of Interest

The authors declare that the research was conducted in the absence of any commercial or financial relationships that could be construed as a potential conflict of interest.

## Publisher’s Note

All claims expressed in this article are solely those of the authors and do not necessarily represent those of their affiliated organizations, or those of the publisher, the editors and the reviewers. Any product that may be evaluated in this article, or claim that may be made by its manufacturer, is not guaranteed or endorsed by the publisher.
